# Interpretable predictive model for shield attitude control performance based on XGboost and SHAP

**DOI:** 10.1038/s41598-022-22948-w

**Published:** 2022-10-29

**Authors:** Min Hu, Haolan Zhang, Bingjian Wu, Gang Li, Li Zhou

**Affiliations:** 1 SILC BusinessSchool, Shanghai University, Shanghai, 201800 China; 2 SHU-SUCG Research Centre for Building Industrialization, Shanghai University, Shanghai, 200072 China; 3 Shanghai Tunnel Engineering Co., Ltd, Shanghai, 200127 China

**Keywords:** Engineering, Civil engineering

## Abstract

The sudden decline in the attitude control performance is a common abnormal situation during shield tunneling. When the problem happens, the shield driver will have difficulty controlling the shield's attitude, which will cause the shield to deviate from its design axis and affect the quality of the tunnel. The causes behind poor control performance are usually complicated, so how to choose appropriate countermeasures is a challenging problem. Based on the above issues, this paper proposes the Interpretable Predictive Model for Shield attitude Control Performance (IPM_SCP). The model first predicts the current shield control performance through the extreme gradient boosting (XGBoost) sub-model and then uses the Shapley additive explanation sub-model to interpret the model output. The model was tested on the left-line tunnel of the Hangzhou–Shaoxing railway project in the Ke-Feng section. The results reveal that the model could effectively predict the control performance of the shield and give the most influential parameter and the direction in adjusting the parameter to improve the shield's attitude control performance when the control performance decreases. Therefore, IPM_SCP gives the correct parameter adjustment instructions when the shield’s attitude control performance declines, and eventually improves tunnel construction quality and efficiency.

## Introduction

The shield tunneling method is widely used in tunneling projects owing to its fast excavation speed, high degree of automation, and low impact on the surrounding environment. However, shield tunneling still faces many challenges; posture instability and tunneling misalignment are two common construction risks during tunneling. Posture instability means that the attitude of the shield cannot be kept to the control target, and tunneling misalignment means that the position of the shield machine deviates significantly from the design axis. In most circumstances, shield misalignment is caused by posture instability. Excessive misalignment can cause segment cracking, segment offset, and even water leakage^1^. For example, in a subway project in Nanning, the shield had difficulty lowering its head, which led to the segments’ dislocation and damage and seriously affected the tunnel quality^2^. Another example is that during the construction of a high-pressure underwater subway tunnel, the vertical deviation of the shield machine was too large due to improper handling of its attitude, which prevented the project from progressing properly^3^.

Posture instability will seriously affect the construction progress, increase project cost, and cause severe consequences, such as segment damage, tunnel water leakage, excessive ground settlement, and even tunnel rerouting^4^. There are five main types of posture discord: (1) the shield machine tilts upward uncontrollably, (2) the shield machine tilts down uncontrollably, (3) the shield drifts upward (the position of shield rises as a whole)^5^, (4) the shield body has difficulty turning horizontally in a certain direction, and (5) the rotation angle of the shield is too large.

The main reason for the posture discord is that the driver cannot easily control the posture of the shield by just adjusting regional oil pressure. Therefore, researchers have attempted to use various methods to resolve the problem. Most studies approach the shield attitude control problems in three ways.

The first type of research focuses on shield attitude anomaly analysis in specific tunneling projects. Guan et al. analyzed the shield attitude adjustment of an inclined shaft project^6^. They concluded that the upper and lower limits of shield elevation adjustment would drop as the downhill angle increases. The sliding force was the leading cause of shield tilting down. They also made several suggestions to prevent the shield tilting down. Li analyzed the factors influencing the shield's attitude on soft ground and gave advice on the shield's potential remedies for lousy attitude in Suzhou metro tunnel project^7^. However, these studies are mainly based on the empirical analysis from technicians; the findings are not sufficiently clear and definitive to be of use as practical guidance.

The second type of research approach is to model the process of shield attitude adjustment based on the analysis of the forces acting on the shield. The theoretical method examines the force between the shield and the soil as well as its impact on the shield’s attitude. Alsahly et al. established a load model to represent the interaction between the surrounding soil and the shield body and developed a computational framework that can obtain the change of the shield attitude during excavation^8^. Yue et al. modeled the hydraulic system and built a simulation system to simulate the variation of shield attitude^9^. However, the theoretical method cannot cover most of the influence of various factors because the model is too simplified. When encountering real-world situations, the model's accuracy will be significantly decreased.

The third research type is the data-driven approach. Compared to the theoretical approach, the data-driven approach is based on actual engineering data and uses machine learning algorithms to predict key control parameters. Li et al. used gated recurrent units (GRU), a variant of recurrent neural networks that utilized real-time tunneling parameters to predict the tunneling attitude of the shield^10^. Shen et al. incorporated wavelet transform into a Long short-term memory (LSTM) network to predict the attitude and position of the shield^11^. This type of method improves the accuracy of attitude prediction. However, owing to the model’s lack of interpretability, it is almost impossible to clarify the relationship between the features and the model's output, so it is impractical for on-site personnel to use the model as a guide to solve the problem of deteriorating shield control performance despite the high accuracy the models have. Furthermore, the method can only be applied in specific conventional engineering contexts because most of the methods primarily consider the common situations and omit the unusual ones. Furthermore, once complications occur in shield attitude control, it is not easy to use the models directly to improve the control power of the shield.

From the perspective of controlling the shield, the major manifestation of posture instability is that the shield's attitude control performance does not meet the expectations. Therefore, how to evaluate and improve attitude control performance is a significant issue. However, except for a few studies that have focused on studying shield attitude control under abnormal situations for specific projects^6,7^, most studies emphasize attitude control optimization in the normal state.

At present, there are few studies on shield attitude control performance. Nevertheless, some researchers revolved around the tunneling equipment's excavation performance to improve the shield machine's tunneling capacity. Wang et al. established the rock mass rating (RMR) system and the empirical formulas of the shield tunneling rate, construction progress, utilization rate, and penetration index using the regression method^12^. Avunduk and Copur and Arbabsiar et al. established multiple regression models to predict the thrust, cutter disk torque, and other tunneling parameters of shields under different soil conditions, as well as performance indexes such as instantaneous cutting rate and energy consumption^13,14^. Martins and Miranda and Mahdevari et al. used the artificial neural network and support vector regression (SVR) models to establish a hard rock excavation speed prediction model^15,16^. Fattahi and Babanouri also used the SVR model to predict the excavation rate and used a variety of heuristic algorithms to optimize the parameters of the model^17^.

Inspired by the excavation performance studies, this paper focuses on evaluating, analyzing, and optimizing shield attitude control performance to fill the research gap. We designed an interpretable attitude performance prediction model as well as an index for shield attitude control performance. The model can not only accurately indicate the current shield's attitude control performance with the help of the index, but also can show the effects of the input parameters for the decline of the attitude control performance so that construction personnel can take effective countermeasures eventually.

In cooperative game theory, the Shapley value^18^ is used to evaluate each player's contribution fairly. Suppose we regard each control parameter of the shield as a participant. In this case, we can refer to the Shapley value to quantify the marginal contribution of each control parameter for attitude control to gain a clearer understanding of which control parameters have the most significant impact on the current control performance and how the parameters influence it. Then, the control parameters can be adjusted more precisely. Based on this idea, combined with the extreme gradient boosting (XGBoost) model’s advantage in prediction accuracy, this paper proposes an Interpretable Predictive Model for Shield attitude Control Performance (IPM_SCP).

The rest of the paper is organized as follows. Section 2 summarizes the current research on shield attitude control. Section 3 designs the attitude control performance index from the attitude control mechanism. Section 4 presents the architecture of the IPM_SCP model, and Sect. 5 shows the application of the model in the left-line tunnel of the Hangzhou–Shaoxing railway project in the Ke-Feng section (H–S|K-F Project). Finally, Sect. 6 provides a final summary of the paper.

## Literature review

The shield attitude control studies can be divided into two types: theoretical analysis based and data-driven.

### Theoretical analysis based methods

Methods based on the theoretical analysis establish models that describe the interaction between the shield and the surrounding soil. Shield weight, force upon the shield tail, thrust, excavation surface soil resistance, and shield surface resistance are the key considerations for these models. Sramoon et al. established a shield load model based on the forces mentioned above and analyzed the factors affecting the shield attitude control based on the force balance equation^19^. Similarly, Yue et al. established a kinetic mechanical model and used a numerical simulation to predict the effect of the various attitude correction strategy^20^. Sugimoto et al. demonstrated a finite element model and used it to predict the attitude change of the shield^21^. Yue et al. made a model for shield attitude and the electromagnetic hydraulic system^9^. Accordingly, a jack control strategy used for shield correction was proposed.

This method is also used in the analysis of shield attitude control performance. Guan et al. developed a mathematical model based on the Winkel elastic foundation model and shield load^6^. The model was used to describe the shield attitude adjustment ability under inclined shaft conditions. He concluded that the greater the downhill angle (the sliding force of the shield), the lower the bottom limits of the shield's vertical adjustment capability. He then gave suggestions on how to prevent the shield from pitching forward too much. Li established a load model and analyzed the influence factors of the shield’s attitude in the soft soil layer through simulation, and then gave recommendations on the attitude correction strategies of shield^7^.

In summary, while methods developed from the theoretical analysis have great interpretability and can reflect the key factors that affect attitude control, the generalization of the model is usually poor because these models rely heavily on assumptions of the forces, soil descriptions, and parameter settings. Therefore, it is difficult to directly guide the tunnel construction with these models.

### Data-driven method

With the rise of machine learning, more researchers have chosen data-driven approaches to study the attitude control of shields. These methods use the shield parameters as the input of the model and the deviation of the shield posture as the model output. SAKAI and HOSHIYA applied the Kalman filter theory to the attitude control of the shield and established an autoregressive predictive control model^22^. Xia et al. and Wang et al. used the XGBoost model to predict the deviation of the shield^23,24^. The model's input is composed of control parameters such as cutter speed, cutter torque, total thrust, and advance speed. Then, the control parameters are optimized based on experience and rules if the model output yields poor results. Many researchers have also used deep learning models on shield attitude prediction. Zhou et al. used the combination of convolutional neural networks and LSTM networks, that is, the CNN + LSTM method, to extract the features of the time series of the shield parameters and predict the attitude deviation^25^. Hu et al., Li et al., and Zhang et al. used recurrent neural networks to predict the attitude deviation^10,26,27^. Data-driven methods can reflect the correlation characteristics of the parameters and have high short-term prediction accuracy, but they are prone to overfitting. At the same time, machine learning models lack interpretability and cannot explain the relationship between the model input and output. Therefore, these models are only used for attitude prediction instead of control attitude directly.

## Summary

The traditional modeling method based on the theoretical analysis has fewer factors, and its model parameters are hard to obtain, which causes its weak prediction performance. In contrast, data-driven modeling methods used in attitude deviation prediction have the advantage of high accuracy. However, owing to their lack of interpretability, it is difficult to use them in attitude control because they still depend on experience and rules for the on-site workers to select a control strategy when the shield encounters abnormal conditions. Although some researchers have studied the causes for the difficulty of shield attitude control, there are hardly any universal index for shield attitude control performance and these studies have often been confined to a specific project or stratum condition, so their conclusions can hardly be applied to other projects.

Therefore, this paper aims to design a universal index that can indicate shield attitude control performance and identify a model that is both accurate and interpretable to analyze the influence of various shield parameters related to attitude control performance and the interaction between parameters. In this way, the model can genuinely guide the shield attitude control as the model can provide direction in adjusting specific parameters to improve the attitude control performance of the shield when the attitude control performance is weak.

## Shield attitude control performance index

### Attitude control principal analysis

**Figure 1:**
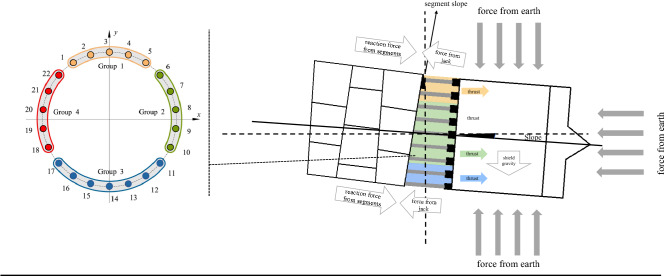
Shield Attitude Control Mechanics.

The shield attitude is mainly controlled by the jacks. To simplify the control process, jacks are grouped according to the diameter of the shield, usually forming four or six groups. The shield driver controls the shield posture by adjusting the oil pressure of each group. Take the four-group shield machine as an example. The way the jacks are grouped is shown in Fig. 1. In theory, when the shield needs to lift upwards, the shield driver needs to increase the oil pressure in the lower group and reduce the oil pressure in the upper group. When the shield needs to turn left, the shield driver needs to increase the oil pressure in the right group, reduce the oil pressure in the left group, and vice versa. The oil pressure is controlled by adjusting the valve opening of the jacks. This is the fundamental way to control the attitude of the shield. However, this is not necessarily the case in the real world. As the thrust system of the shield is a hydraulic system, its actual oil pressure not only depends on the valve opening but is also related to the surrounding load, such as the geotechnical condition and control related features like the pressure of the front soil on the cutter, the slope of the shield, and the force from the rear segment are all contributing factors. The complexity of the shield's excavation situation has led to the phenomenon where the attitude cannot be entirely in control with only the valve opening adjustment during the construction^28^. This phenomenon is also the direct cause of most posture instability and misalignment problems.

### Shield attitude control performance index design

Considering the intricacy of the shield's attitude control performance, how to evaluate the control performance is challenging. Currently, most similar indexes are specific to their problems and lack universality. For instance, Guan et al. proposed an index to evaluate the shield's ability to pitch up using the normalized settlement difference between the tunnel head and tunnel tail^6^. This index is only suitable for inclined shaft tunneling. Therefore, to improve the universality and accurately reflect the shield's control performance, we introduce a shield control performance index based on the way shields are controlled. We use the ratio of the oil pressure difference (the same direction to the attitude change) to the shield attitude change as the Shield Attitude Control Performance Index (SCPI) as shown in Eq. (1)

1 $$SCPI_{i} = { }\Delta q_{i} /\left( {\Delta p_{i} + 1} \right)$$

Where, *i* is the sample number; $\Delta q_{i}$ is the ith horizontal or vertical oil pressure difference, based on the direction of the attitude variance; and $\Delta p_{i}$ is the ith horizontal or vertical angle variance. The one that is added to $\Delta p$ prevents the denominator from being zero. The basic principle behind this index is that when the shield attitude control performance is poor, it requires a much more significant oil pressure difference to achieve the same amount of attitude movement.

To make the results easier to understand, we map the results obtained by the above calculations to the interval from 0 to 100, as shown in Eq. (2).

2 $$score_{i} { } = { } - 100\cdot\left| { - 1 + \frac{{2(SCPI_{i} - SCPI_{min} )}}{{SCPI_{max} - SCPI_{min} }}} \right| + 100$$

The closer the score is to 100, the better the control performance. Generally, the score should be greater than 60 under most circumstances. If it is less than 60 points or drops suddenly, there is a problem with the attitude, which requires attention. If the attitude correction is not in line with the expected effect, specific countermeasures should be taken immediately.

## Interpretable predictive model for shield attitude control performance

### Model framework

**Figure 2:**
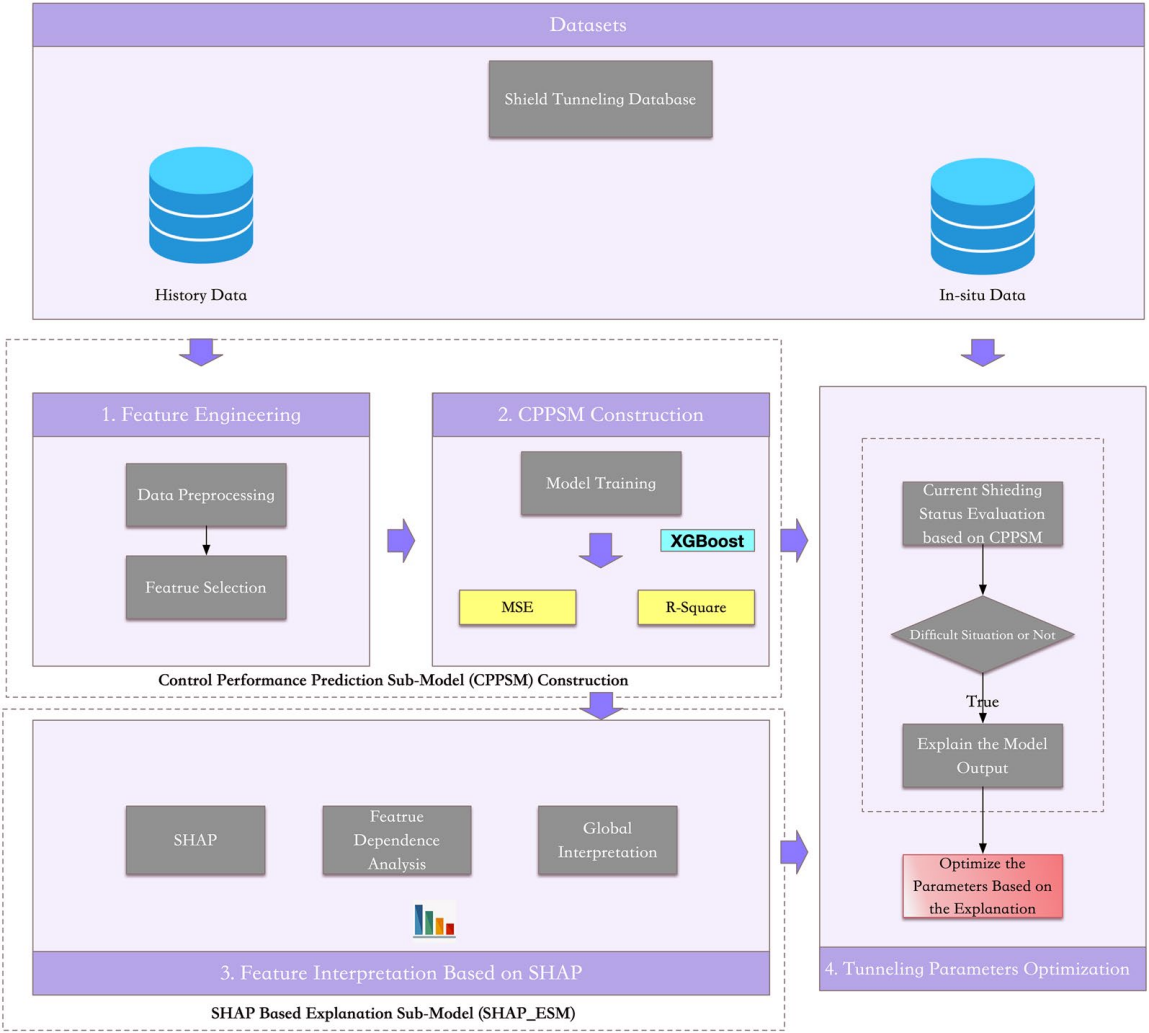
IPM_SCP framework.

To improve the interpretability of the model so that it can provide support for formulating attitude control strategies, we designed an Interpretable Predictive Model for Shield attitude Control Performance (IPM_SCP), which combines XGBoost^29^ and Shapley additive explanation^30^. IPM_SCP is a model that describes the relationship between each input control parameter and the model output. The idea behind the model is from game theory. After analyzing the influence of each input control parameter, the key control parameters for performance degradation can be found. In IPM_SCP, the impact of each input control parameter of every sample can be quantified. The overall framework of the model is shown in Fig. 2.

IPM_SCP is divided into four steps: (1) Preprocess the shield tunneling data according to the attitude control and feature independence principle; (2) Use historical data to train a predictive model for predicting SCPI based on the model input and output; (3) Based on the shield attitude control performance prediction model, use SHAP to analyze the relationship between input features and the model output as well as the magnitude and direction of the influence of the features; (4) If the current attitude control performance is weak, analyze each feature's importance and direction of its influence with the model and the in situ data, formulate adjustment strategies, and optimize the control parameters to improve the attitude control performance.

### Input feature selection

**Figure 3:**
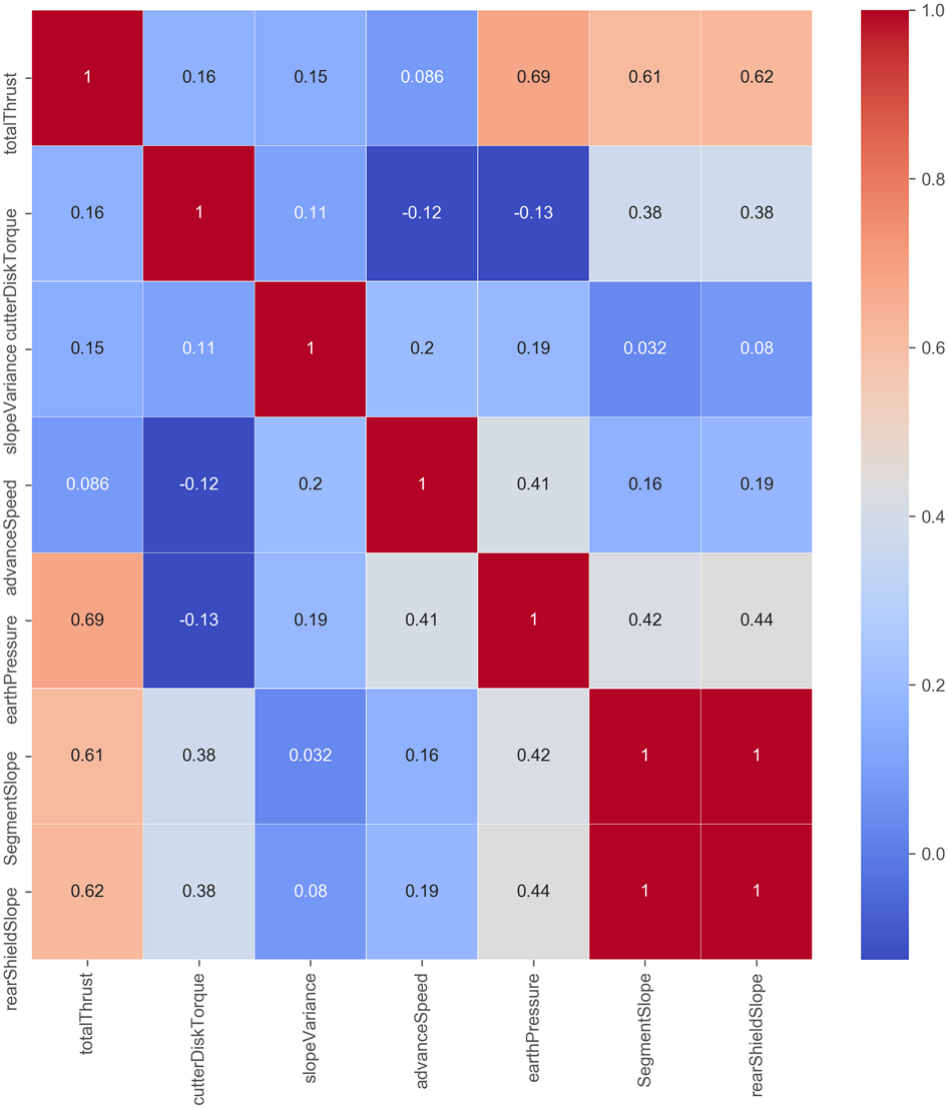
Feature Correlation Analysis.

Based on the shield attitude control performance index, we conduct a preliminary selection of the input features of CPPSM. The features crucial to shield control performance according to Sect. 3.1 can be categorized into two kinds, namely geotechnical condition features and control related features. Because the goal of IPM_SCP is to improve the control performance of the shield by providing direction in adjusting the control parameters, and the geotechnical condition is immutable, thus we do not put these features in our model. Instead, we treat these features as background information. Therefore, the candidate features mainly come from control parameters which include the total thrust, advance speed, cutter disk torque, earth pressure, rear shield slope, segment slope, and slope variance (Table 1). To ensure the effect of the model, we calculate the linear correlation coefficient between each feature (Fig. 3) and eliminate the features that are too similar.

**Table 1:** Input feature description and feature selection results.

Input features	Feature description	Feature selection
Total thrust	The total thrust of the jacks during tunneling. It reflects the load of the shield (KN)	√
Advance speed	The speed of the shield when tunneling (mm/min)	√
Cutter disk torque	The torque of the Cutter disk when spinning (N˙m)	√
Earth pressure	The value of the earth pressure sensor in the soil compartment (bar)	√
Shield rear slope	The value of the angle sensor of the rear shield (°)	√
Segment slope	The slope of the last segmental ring (°)	×
Slope variance	The variance of the shield rear slope within an interval	√

Figure 3 shows that the segment slope (the shield slope at the end of the last segment assembly) is highly related to the rear shield slope. Therefore, this paper selects the rear shield slope as the model input instead of the segment slope. The results of the feature selection are shown in Table 1.

### Control performance predictive sub-model (CPPSM)

Because the XGBoost algorithm can achieve better accuracy in multiple regression tasks and is not easily prone to overfitting^29^, this paper uses the XGBoost algorithm to establish the relationship model between the shield control performance and shield attitude. The input feature of the model is the feature set selected in Table 1, and the output is the SCPI (see Eq. (1)). The predicted output value of the model is the sum of K decision trees. The specific equation is shown in Eq. (3).

3 $$\hat{y}_{i} = \mathop \sum \limits_{k = 1}^{K} f_{k} \left( {{\text{x}}_{i} } \right),f_{k} \in F,$$

Where, $\hat{y}_{i}$ is the model output of ith sample i, which is the predicted attitude control performance;${\mathbf{x}}_{i}$ is the ith model input; K is the number of decision trees;$f_{k} \left( {{\mathbf{x}}_{i} } \right)$ represents the result of the kth decision tree; and F is the space of every tree.

The objective function of the model is shown in Eq. (4).

4 $$\left\{ {\begin{array}{lllll} {Min L\left( \phi \right)} \\ { L\left( \phi \right) = \mathop \sum \limits_{i} l\left( {\hat{y}_{i} , y_{i} } \right) + \mathop \sum \limits_{k} \Omega \left( {f_{k} } \right)} \\ {l\left( \theta \right) = \mathop \sum \limits_{i} \left( {y_{i} - \hat{y}_{i} } \right)^{2} / n} \\ {\Omega \left( {f_{k} } \right) = \gamma T + \frac{1}{2}\lambda \mathop \sum \limits_{j = 1}^{T} w_{j}^{2} } \\ \end{array} } \right.$$

Equation (4) is composed of the loss function, $l,$ and the regularization term, ${\Omega }$. The former depicts the accuracy, while the latter controls the complexity of the model. Here, n is the total number of samples;$\hat{y}_{i}$ is the ith model's output;$y_{i}$ is the ith ground truth; T represents the number of leaves;$w_{j}$ is the value of the jth leaf nodes; $\gamma$ and $\lambda$ are tuneable parameters, where a larger value of $\gamma$ and $\lambda$ indicates simpler tree structure.

### Explainable Sub-model based on SHAP (SHAP_ESM)

There are several ways of getting the feature importance from an XGBoost model. For instance, based on the basic decision-tree structure of the XGBoost model, one can sum up the gain of each feature split on each node^31–33^ and count how many splits in the decision tree originated from the same feature. However, these methods may have inconsistencies ^30,34^, which makes the result unreliable. Also, these methods cannot reflect the direction of each feature influence on the model output, nor can they reflect the interaction between features.

SHAP provides a path for in-depth feature analysis and interpretation^34^. It regards the features in the machine learning model as participants in Shapley, and the predicted value as the goal of all participants. By calculating the Shapley value of each feature, the contribution of each feature to the predicted value can be calculated. Based on the principle of SHAP, the output of CPPSM can be obtained by adding the contribution value of each feature, as shown in Eq. (5).

5 $$g\left( {z^{\prime}} \right) = \phi_{0} + \mathop \sum \limits_{m = 1}^{M} \phi_{m}$$

Where, $\phi_{0}$ is the base value of the model, namely the mean value of all model outputs.

$\phi_{m}$ is the *m*th feature’s SHAP value, and *M* is the number of features.

Equation (6) shows the calculation detail of the contribution of feature m $\phi_{m}$. The idea is that each feature subset contains feature *m* in a particular order. The model output with and without feature *m* of each feature subset is calculated first. The difference between the two is multiplied by the probability of occurrence of the subset. Then, the marginal contribution of feature *m* can be obtained under this feature subset. The final contribution of feature *m* is obtained by accumulating the marginal contributions of all feature subsets that contain feature m. A positive contribution value indicates that the feature positively affects the model output, and a negative value indicates that the feature negatively affects the model output.

6 $$\phi_{m} = \mathop \sum \limits_{{S \subseteq N\left\{ m \right\}}} \left| S \right|!\left( {M - \left| S \right| - 1} \right)! \left[ {v\left( {S \cup \left\{ m \right\}} \right) - v\left( S \right)} \right]/M!$$

$${\raise0.7ex\hbox{${S \subseteq \left\{ {x_{1} ,x_{2} , \ldots x_{M} } \right\}}$} \!\mathord{\left/ {\vphantom {{S \subseteq \left\{ {x_{1} ,x_{2} , \ldots x_{M} } \right\}} {x_{m} }}}\right.\kern-\nulldelimiterspace} \!\lower0.7ex\hbox{${x_{m} }$}}$$

Where, $v\left( S \right)$ is the model output without feature $m$.

## Engineering application

### Engineering background

**Figure 4:**
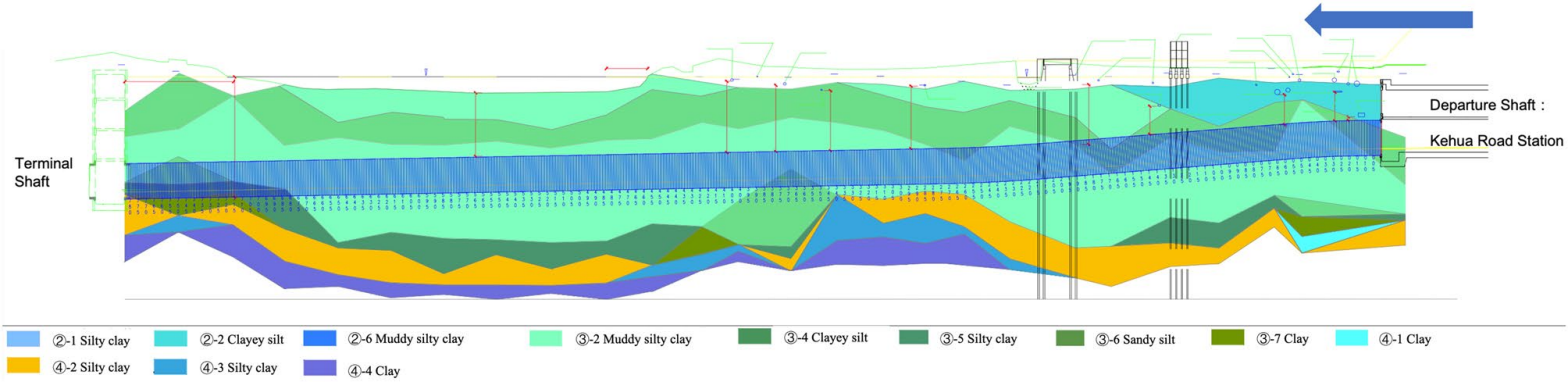
Geological Description of the Left Line Tunnel of Ke-Feng Section.

During the excavation of the left-line tunnel of H–S|K-F Project with *ZhiYu* self-driving shield, we applied the IPM_SCP model. The model provided suggestions for the control parameter adjustments of the self-driving shield attitude control model. The soil quality in this section is mainly silty clay, as shown in the geological profile (Fig. 4). The characteristics of the soft soil in this area include high moisture content, high compressibility, low strength, and low water permeability. It was challenging to control the attitude of the shield owing to these properties of the soil. In addition, because the tunnel would excavate about 200 rings at the bottom of the Daban Lake, the interaction between the shield and the surrounding soil could not be monitored by ground settlement monitoring or other traditional measures. This made it hard to evaluate whether the control parameters were properly set. During the tunneling process, the shield encountered two difficult conditions where it had difficulty raising its head position. With the help of IPM_SCP to evaluate the shield control performance and feature analysis, the shield adjusted the control parameters according to the model in time. Finally, we overcame the situation and achieved good excavation results.

### Modeling with H–S|K-F project data

**Figure 5:**
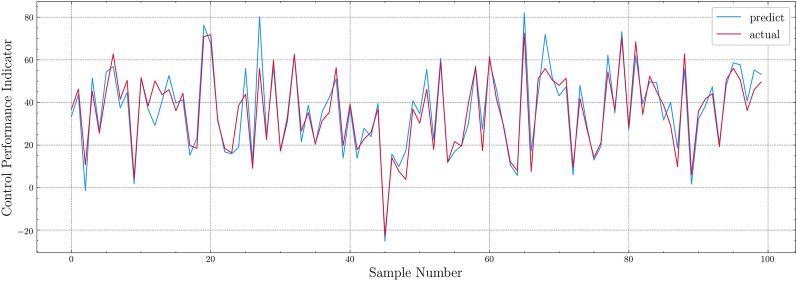
CPPSM testing result.

To train the CPPSM, we use the millimeter-level tunneling data of the right line of the H–S|K-F Project, which have similar characteristics to the left line. The data are randomly divided into the training set and test set by the ratio of 4:1. The sub-model is trained and verified by tenfold cross-validation. The performance of the model is measured with R-square and MSE (Table 2). Figure 5 compares the predicted value and the actual value of the model for 100 samples in the test set. Considering the 10-mm measurement error of the shield attitude measurement system itself, this prediction model can meet the basic requirements of attitude prediction.

**Table 2:** CPPSM training result.

Training set R^2^	Training set MSE	Test set R^2^	Test set MSE
0.861	6.81	0.753	9.03

### Feature attribution analysis

According to the results from CPPSM, we can analyze their influence on the shield attitude control performance by calculating the SHAP value of the features and plotting figures that depict the feature contributions and feature dependence.

#### (1) Impact analysis of single feature

**Figure 6:**
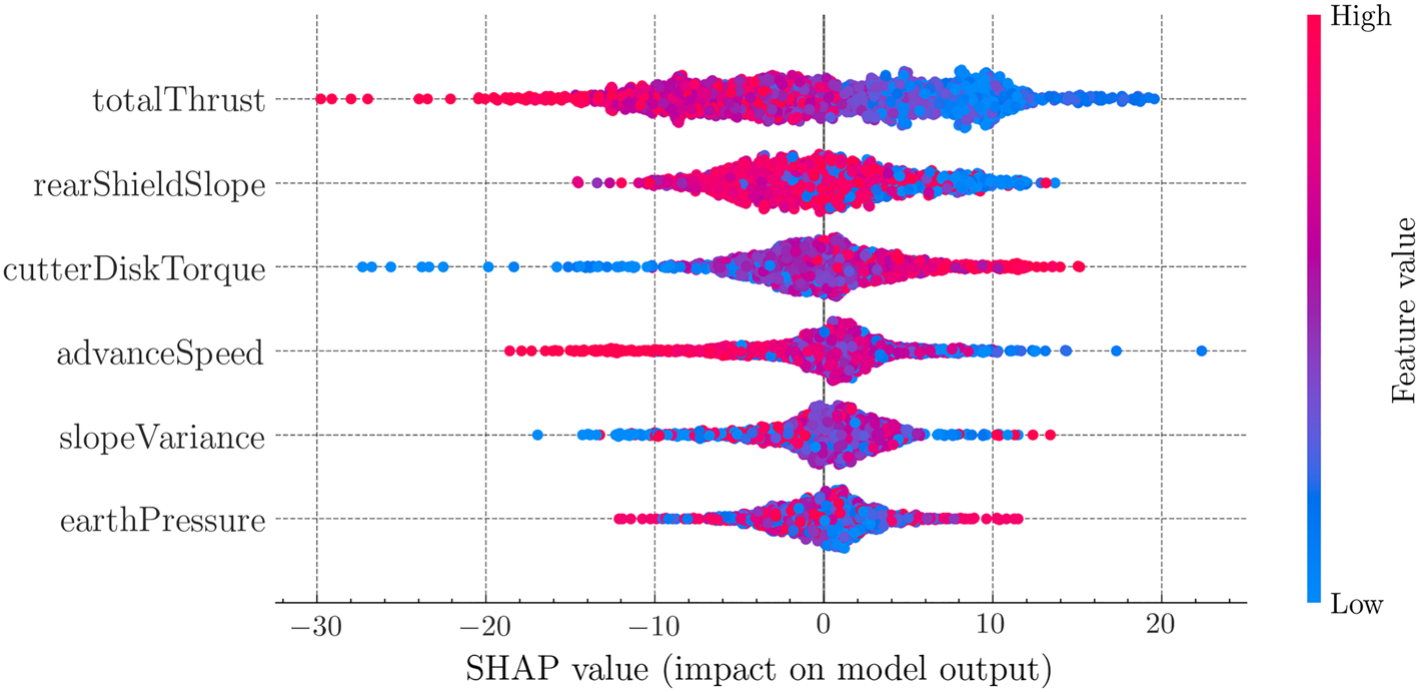
SHAP summary plot.

The summary scatter plot is shown in Fig. 6. Each scatter point represents the SHAP value of a specific feature from a sample. The SHAP value can be regarded as contribution value, so each row of scatter points shows the contribution of a single feature on the attitude control performance. The feature importance is ranked according to the average absolute value of SHAP values. The coverage of the points reflects the degree of aggregation of the sample feature value, and the color of the points indicates the feature value itself. It can be inferred that the color transition of the total thrust is smooth, which means its influence on attitude control performance is inversely proportional to its value. The larger the total thrust value, the smaller its contribution value. Similarly, the values of advancing speed and rear shield slope are inversely proportional to their contribution values, and the cutter disk torque is proportional to their contribution values.

**Figure 7:**
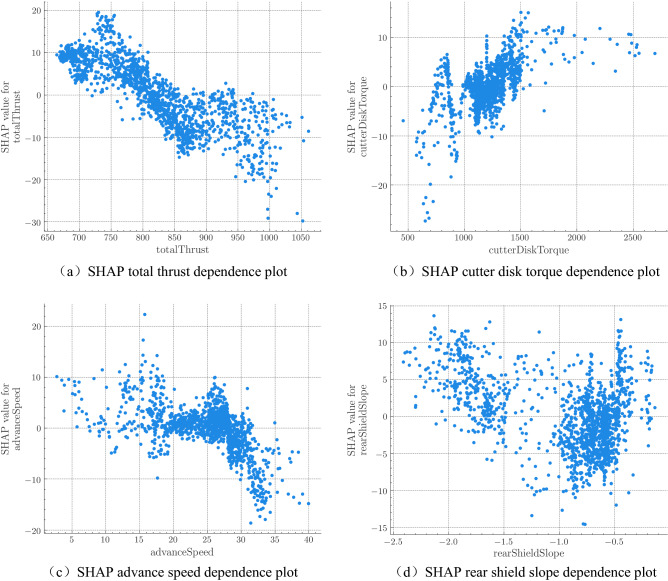
SHAP feature dependence plot: (**a**) SHAP total thrust dependence plot; (**b**) SHAP cutter disk torque dependence plot; (**c**) SHAP advance speed dependence plot; (**d**) SHAP rear shield slope dependence plot.

In order to further analyze the traits of the features, we draw partial dependence plots (Fig. 6) to explore the how the feature contribution changes with the feature value. In the figure, the x-axis is the value of the feature, and the y-axis is the SHAP value of the feature. Figure 7(a) indicates that the total thrust has a negative effect on the attitude control performance. In Fig. 7(b), cutter disk torque is mostly between 1000 and 1500 KNm, and its contribution value initially increases along with the cutter head torque. When its value is greater than 1400 KNm, its contribution tends to stabilize. The value of the advance speed in Fig. 7(c) is generally inversely proportional to its contribution value. When the advance speed is greater than 27 mm/min, the impact on the contribution value increases significantly. In Fig. 7(d), the rear shield slope is between −2.5 and −1.5, and the influence on the contribution value is negatively correlated. When it is greater than −1, the contribution is more scattered positive, in correlation with its value.

#### (2) Dependence analysis of double features

**Figure 8:**
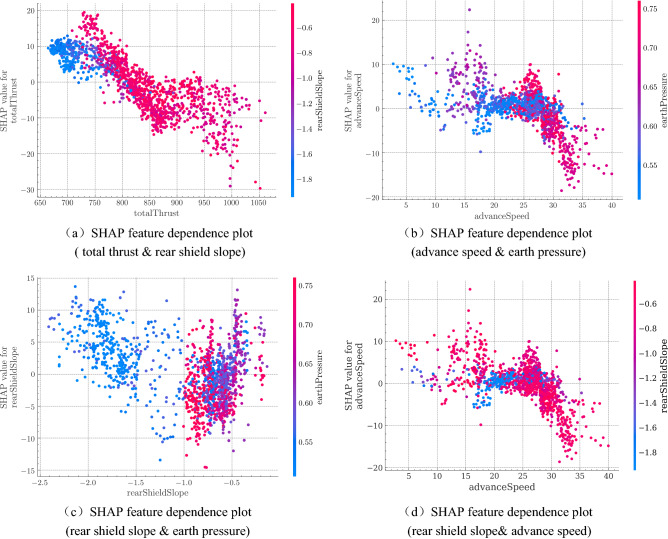
SHAP double feature dependence plots: (**a**) SHAP feature dependence plot (total thrust & rear shield slope); (**b**) SHAP feature dependence plot (advance speed & earth pressure); (**c**) SHAP feature dependence plot (rear shield slope& earth pressure); (**d**) SHAP feature dependence plot (rear shield slope& advance speed).

The contribution of a feature depends on another feature in many cases. Therefore, we draw double feature partial dependence plots (Fig. 8) to show characteristics between the features as well as the distribution traits. The x-axis of the dual feature dependency plot indicates the value of the main feature; the y-axis indicates the SHAP value of this feature; and the color of the point represents the value of the second feature.

Figure 8(a) shows the interaction between the thrust and rear shield slope. The thrust has an obvious interaction with the rear shield slope in the range of 700–750 KN. The overall thrust of this interval increases the control performance of the shield. When the thrust is the same in this interval, the smaller the rear shield slope, the less the contribution of the thrust to the control performance, indicating that the impact of the rear shield slope in this interval may be greater than the impact of the thrust.

Figure 8(b) shows the relationship between advance speed and earth pressure. It can be inferred that in the interval of 25–28 mm/min, the lower the value of earth pressure, the greater the contribution of advance speed to the control performance. In the interval of 28 to 40 mm/min, the value of earth pressure is neither too big nor too small. However, in this interval, the change of the contribution value of the advance speed is reduced faster, so it can be inferred that the earth pressure affects the rate of change of the advance speed's contribution.

Figure 8(c) shows the interaction between the rear shield slope and the earth pressure. When the value of the earth pressure is low, the value of the slope is also low, mostly less than −1, and the slope contribution value in this interval has an inverse relationship with its value. When the value of the earth pressure is high, the value of the slope is mostly greater than −1, and the relationship between the slope contribution value and its value in this section is no longer in reverse and instead has a positive trend. Therefore, it can be inferred that the increase in the earth pressure affects the contribution of the rear shield slope.

Lastly, figure 8(d) depicts the mutual effect between rear shield slope and advance speed. The interaction mainly takes place in the speed interval of 15 to 20 mm/min. Specifically, when the rear shield slope has a larger value, roughly above − 0.8 degrees, the contribution of the advance speed seems to get more significant. Conversely, the contribution becomes weaker when the rear shield slope is below − 1.4 degrees. This means that a bigger rear shield slope may favor the shield's control performance during slow excavation.

In summary, when the control parameters are being adjusted, it is crucial to consider the interaction between the features instead of focusing on the influence of a single feature so that attitude control performance can be improved more appropriately.

### Attitude control performance optimization

#### 319th–329th ring

**Figure 9:**
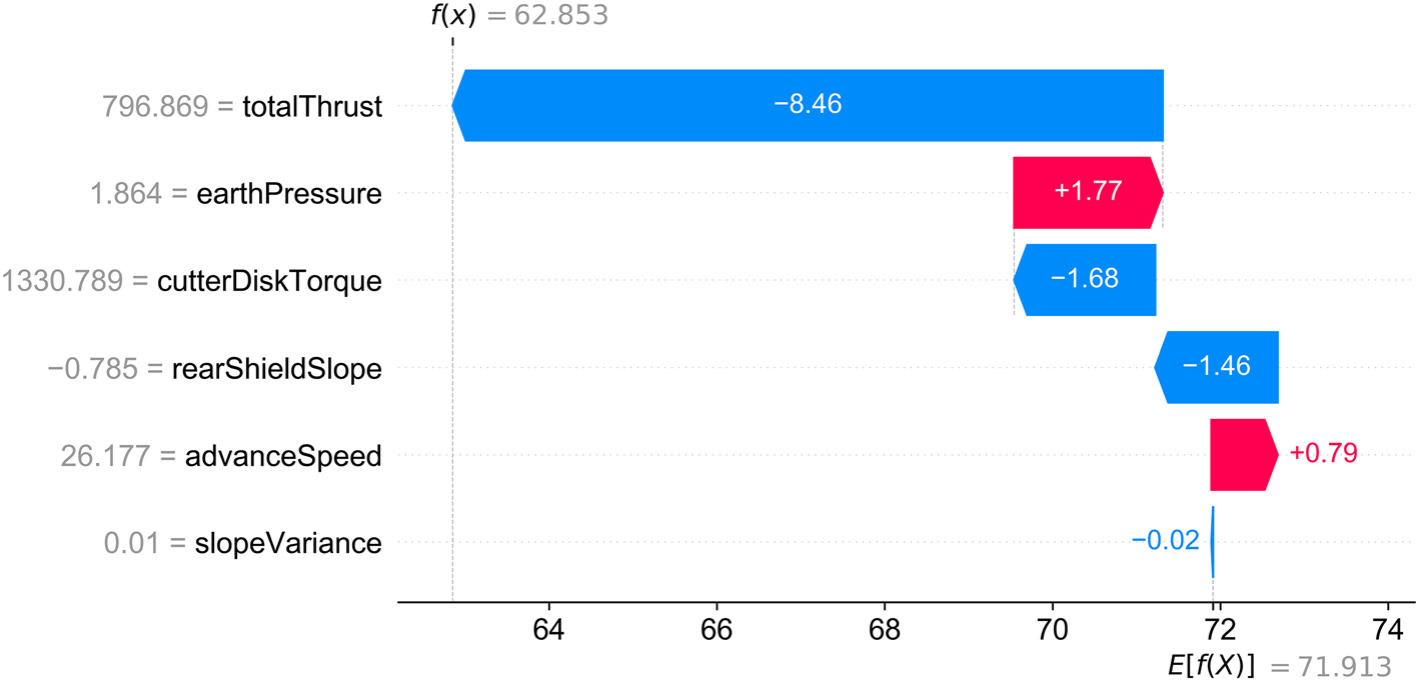
Explanation Result of the 319th ring.

When the shield machine advanced to the 319th ring, the on-site personnel and the model itself found that the shield could not raise its head effectively according to the control target even though the oil pressure difference between the upper and lower groups of the jacks was already huge. At this time, the attitude control performance score was 62.8, and the score of the 312th ring was 89, so the vertical attitude control performance of the shield dropped significantly in just seven rings. For this reason, the autonomous shield attitude control model used IPM_SCP to analyze the data near the 319th ring. Figure 9 displays the contribution of each feature and shows that the total thrust has the most significant influence on the model's output, followed by earth pressure and cutter disk torque. Because the thrust and cutter disk torque are the parameters that mainly reflect the external load, and the advance speed is already very high, the model recommended raising the earth pressure value according to the direction instead.

**Figure 10:**
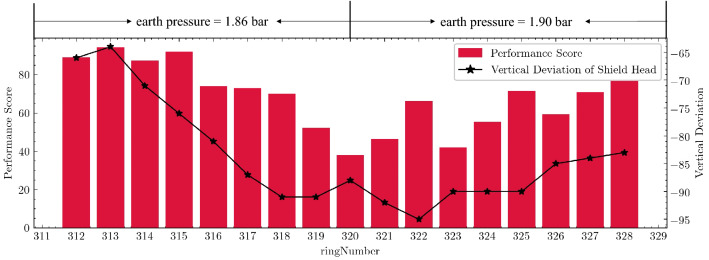
The trend of vertical deviation and performance score before and after adjusting earth pressure (ring 312–328).

As the earth pressure rose from the original 1.86 bar to the 1.90 bar implied by Fig. 10, the shield's ability to lift its head was significantly improved after the 320th ring. The shield attitude control performance index score returned to more than 80 points gradually after the 323rd ring, which showed that the control performance of the shield was significantly improved. This effect was also reflected in the vertical deviation of the shield's cutter, as the position of the shield rose considerably at the 322ed ring (the closer the vertical deviation is to zero, the better). The results show that the strategy given by the model worked.

#### 642ed–651st ring

**Figure 11:**
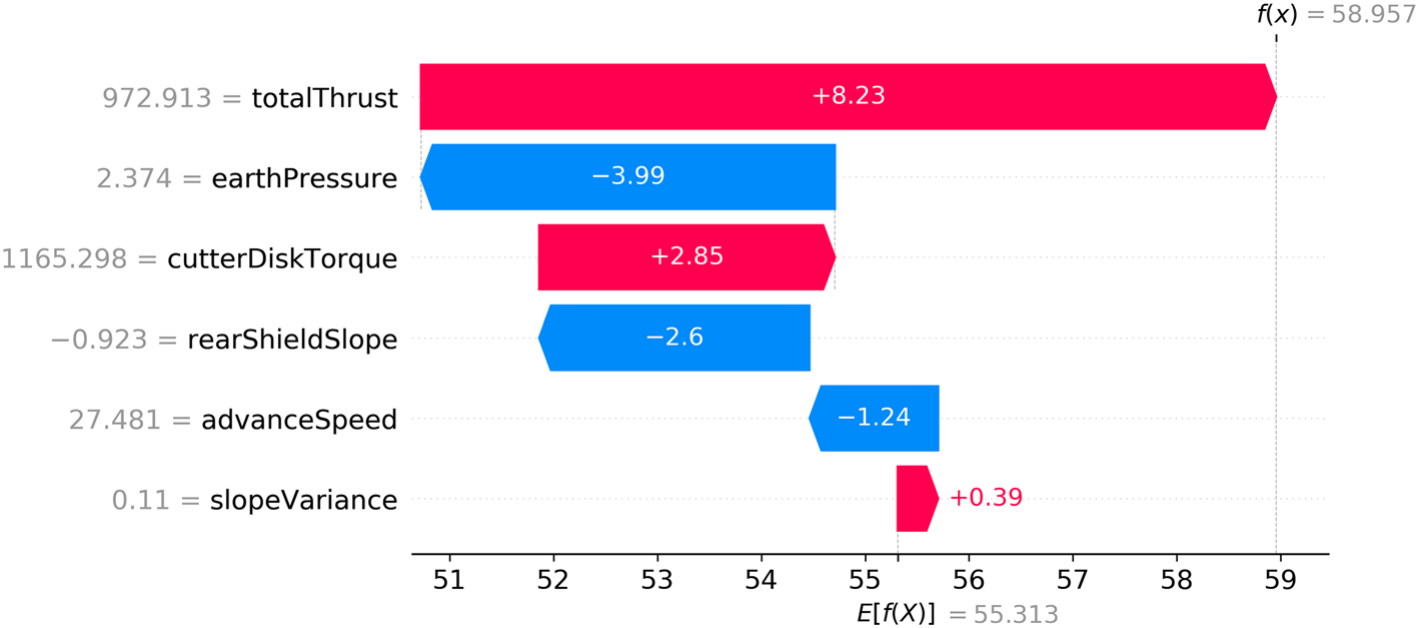
The Explanation Result of the 642ed ring.

When the shield advanced to the 642ed ring, the shield had trouble lifting the head position again. The control performance score of this ring was 59, which was 20 points less than the score for the earlier ten rings. Figure 11 shows the contribution of each feature of the 642ed ring given by the model. The total thrust and earth pressure have the greatest impact according to the model. As the total thrust is determined by the advance speed and external load, the earth pressure is still the focus of this strategic adjustment. However, unlike the 319th ring, the earth pressure was negatively correlated with the contribution degree at this time.

**Figure 12:**
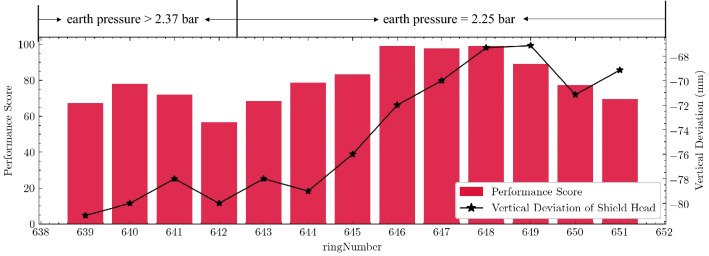
The trend of vertical deviation and performance score before and after adjusting earth pressure (ring 639–651).

Therefore, the self-driving shield control model reduced the earth pressure from 2.37 bar to 2.25 bar. Figure 12 shows the change in the actual control performance of the shield attitude after the earth pressure is adjusted. After the earth pressure had been adjusted, the score improved significantly. In the next few rings, the vertical attitude control performance was also continuously improved, from 59 points to almost 100. The vertical deviation of the shield's cutter also gradually rose. Therefore, the shield attitude was significantly improved.

## Conclusion

This paper establishes an interpretable predictive model for shield attitude control (IPM_SCP). This model combines the XGBoost algorithm and SHAP to predict a shield attitude control performance. It takes advantage of the SHAP value to determine the contribution of each input control parameter for the attitude performance, while XGBoost predicts the actual control performance with precision. The results can accurately reflect the influence that the various control parameters have on the attitude control performance. The model also provides a basis for the formulation of attitude control strategy for the shield. This method was applied to the left line of H–S Project, where it successfully solved two attitude control issues during the tunneling. Although the symptoms of two occasions both appeared to have difficulty raising the head position, two opposite methods of adjusting earth pressure were applied for the shield machine based on the explanatory analysis of the model, which both achieved good results and successfully got rid of the engineering dilemma. Therefore, applying the explanatory model can help accurately grasp the real cause of poor posture control so that personnel can adopt precise control strategies, avoid making decisions blindly, and improve construction quality and efficiency.

IPM_SCP emphasizes nonconventional attitude control and focuses on shield control performance. It also proposes the shield attitude control performance index and an interpretable shield control performance prediction system. It determines the adjustment direction of each control parameter by analyzing the interactive relationship among all the input control parameters as well as their influence on shield control performance. Compared with other methods, IPM_SCP can quickly find out the factors that cause abnormality when the shield control is poor so as to guide the excavation of the shield better.

Admittedly, although IPM_SCP achieved some degree of success in practical applications, it still has limitations. For example, the interpretability of IPM_SCP is highly dependent on the accuracy of its sub-models. However, because of the complex conditions of the tunneling environment, it is necessary to retrain the model when a new project starts. Lack of data at the beginning of a project will affect model performance. Therefore, in future research, we need to make better use of the trained model by using transfer learning or other methods. If the model can achieve better results on a smaller dataset, the reliability of interpretation will be further improved, and the model will also have a broader application.

## Data Availability

The datasets generated during and/or analyzed during the current study are not publicly available but are available from the corresponding author on reasonable request.
